# The Molecular Mechanisms That Underlie the Immune Biology of Anti-drug Antibody Formation Following Treatment With Monoclonal Antibodies

**DOI:** 10.3389/fimmu.2020.01951

**Published:** 2020-08-18

**Authors:** Anna Vaisman-Mentesh, Matias Gutierrez-Gonzalez, Brandon J. DeKosky, Yariv Wine

**Affiliations:** ^1^George S. Wise Faculty of Life Sciences, School of Molecular Cell Biology and Biotechnology, Tel Aviv University, Tel Aviv, Israel; ^2^Department of Pharmaceutical Chemistry, The University of Kansas, Lawrence, KS, United States; ^3^Department of Chemical and Petroleum Engineering, The University of Kansas, Lawrence, KS, United States

**Keywords:** monoclonal antibodies, anti-drug antibodies, immune response, immunogenicity, neutralizing antibodies

## Abstract

Monoclonal antibodies (mAbs) are a crucial asset for human health and modern medicine, however, the repeated administration of mAbs can be highly immunogenic. Drug immunogenicity manifests in the generation of anti-drug antibodies (ADAs), and some mAbs show immunogenicity in up to 70% of patients. ADAs can alter a drug’s pharmacokinetic and pharmacodynamic properties, reducing drug efficacy. In more severe cases, ADAs can neutralize the drug’s therapeutic effects or cause severe adverse events to the patient. While some contributing factors to ADA formation are known, the molecular mechanisms of how therapeutic mAbs elicit ADAs are not completely clear. Accurate ADA detection is necessary to provide clinicians with sufficient information for patient monitoring and clinical intervention. However, ADA assays present unique challenges because both the analyte and antigen are antibodies, so most assays are cumbersome, costly, time consuming, and lack standardization. This review will discuss aspects related to ADA formation following mAb drug administration. First, we will provide an overview of the prevalence of ADA formation and the available diagnostic tools for their detection. Next, we will review studies that support possible molecular mechanisms causing the formation of ADA. Finally, we will summarize recent approaches used to decrease the propensity of mAbs to induce ADAs.

## Introduction

In the last three decades, the pharmaceutical industry experienced a massive shift toward the use of protein drugs, often referred to as “biologics.” Biologics offer higher specificity and better characterized mechanisms of action compared to small molecule drugs, and their use has revolutionized the treatment of a wide range of diseases and disorders. In general, monoclonal antibodies (mAbs) are the most widely used class of biologics (1).

Monoclonal antibodies account for a growing number of blockbuster drugs with their US sales reaching over $24 billion (2), and will maintain a dominant position in the pharmaceutical market that exceeds $125 billion by the end of 2020 (3).

To date, over 73 mAbs have been approved by the United States Food and Drug Administration (FDA) and the European Medicines Agency (EMA). Hundreds more mAbs are in different stages of clinical developmental. mAbs are used for various clinical indications including cancer, chronic autoimmune and inflammatory diseases, allergies, infections, transplantations, and cardiovascular diseases (4).

The mechanism of action (MOA) of mAbs can vary across different use cases. For example, the anti-CD20 rituximab induces cell death by binding to surface receptors, resulting in a signaling cascade that leads to apoptosis (5). Other mAbs, including the anti-HER-2 trastuzumab, block receptor-ligand interactions to achieve a desired effect, either by blocking the receptor domain to inhibit an activation signal by removing a soluble ligand entirely from circulation (6). mAbs can also induce fragment crystallizable (Fc)-dependent effector functions such as antibody-dependent cell-mediated cytotoxicity (ADCC) and complement-dependent cytotoxicity (CDC), which are important for the anti-CD20 drug obinutuzumab that is used for the treatment of lymphoproliferative disorders (7). Other mAbs target specific proteins involved in pathogenesis of disease, such as anti-TNFα mAbs infliximab and adalimumab that are used to treat inflammatory bowel disease (IBD) and rheumatoid arthritis (RA) (8). Other mAbs in this category are omalizumab, an anti-IgE mAb that is used to treat patients with allergic asthma (9), palivizumab which targets an epitope in the A antigenic site of the F protein of the respiratory syncytial virus (RSV) (10), and bezlotoxumab which binds and neutralizes *Clostridium difficile* toxin B (11). Some mAbs, such as cetuximab and panitumumab (12), target the epidermal growth factor receptor (EGFR) which is overexpressed in a number of cancers. In recent years, checkpoint inhibitor mAbs were also developed to manipulate anti-tumor T-cell responses, like the anti-PD-1 nivolumab that is used to treat melanoma and non-small cell lung cancer (13).

The tremendous progress in mAb discovery began in 1975, when Köhler and Milstein reported *in vitro* screening and production of murine mAbs from hybridomas (14). In the late 1980s, murine mAbs were in rapid clinical development, but had significant drawbacks as they were often induced allergic reactions and the formation of human anti-mouse antibodies (HAMA). Examples include T101 used to treat chronic lymphocytic leukemia (CLL) and cutaneous T cell lymphoma (CTCL), and 9.2.27 to treat melanoma (15). Additionally, murine mAbs exhibited a relatively short half-life in humans, possibly due to low affinity toward the human neonatal Fc receptor (FcRn) (16), and were relatively poor recruiters of effector functions, crucial for some mAb efficacy (17).

To overcome the immunogenicity and reduced effector function of murine mAbs, chimeric antibodies (mouse–human) were next developed by fusing the antigen-specific variable domain of a murine mAb with the constant domains of a human mAb. This resulted in chimeric mAbs of approximately 65% human origin by amino acid content (18). Human gene sequences were mostly taken from the κ light chain and the IgG1 heavy chain, as IgG1 has the highest efficiency in activating complement and cytotoxic effector cells, and the κ light chain is more common in human serum antibodies (19, 20). The development of chimeric mAbs indeed reduced immunogenicity and increased efficacy. For example, metastatic colorectal carcinoma patients who received the chimeric mAb 17-1A did not show any toxic or allergic reactions, and the chimeric antibody was significantly less immunogenic than its parental murine antibody (21).

Chimeric mAbs exhibited an extended half-life and reduced immunogenicity, but they still presented a considerably high propensity for ADA induction (22). Aiming to further reduce mAb immunogenicity, humanized mAbs were developed by grafting the murine complementarity determining regions (CDR) onto framework regions (FR) of the human mAb heavy and light chain variable domains (V_*H*_ and V_*L*_, respectively), for mAbs that are approximately 95% human (23). mAb humanization often significantly reduces immunogenicity and ADA formation (24).

Technological advances of phage display technology (25, 26) based on human single chain Fv (scFv) libraries (27) next enabled the discovery of antibodies comprised entirely of human genes. These human mAbs were additionally aided by the more recent development of transgenic mouse strains expressing human antibody variable domains (28–30).

While both humanized and fully human mAbs reduce immunogenic potential and show properties similar to human endogenous IgGs, they fail to completely eliminate mAb immunogenicity and ADA formation (31). Table 1 summarizes mAbs that are currently approved in the US and EU, along with their reported immunogenicity rates.

**TABLE 1:** Approved mAb and their reported ADA rates.

International non-proprietary name	Brand name	Target	Format	Indication first approved or reviewed	First EU/US approval year	%ADA	%ntADA	References
Adalimumab	Humira	TNFa	Human IgG1	Rheumatoid arthritis	2003/2002	28%	Not reported	(139, 140, 141)
Alemtuzumab	Lemtrada; MabCampath, Campath-1H	CD52	Humanized IgG1	Multiple sclerosis; chronic myeloid leukemia#	2013; 2001#/2014;2001#	67.1–75.4	Not reported	(102, 103)
Alirocumab	Praluent	PCSK9	Human IgG1	High cholesterol	2015/2015	5.1%	1.30%	(142)
Atezolizumab	Tecentriq	PD-L1	Humanized IgG1	Bladder cancer	2017/2016	30–48%	Not reported	https://www.accessdata.fda.gov/drugsatfda_docs/label/2018/761034s010lbl.pdf
Avelumab	Bavencio	PD-L1	Human IgG1	Merkel cell carcinoma	2017/2017	4.10%	Not reported	https://www.accessdata.fda.gov/drugsatfda_docs/label/2018/761069s002lbl.pdf
Basiliximab	Simulect	IL-2R	Chimeric IgG1	Prevention of kidney transplant rejection	1998/1998	1.17%	Not reported	https://www.accessdata.fda.gov/drugsatfda_docs/label/2003/basnov010203LB.htm
Belimumab	Benlysta	BLyS	Human IgG1	Systemic lupus erythematosus	2011/2011	0–4.8%	Not reported	(74)
Benralizumab	Fasenra	IL-5R α	Humanized IgG1	Asthma	2018/2017	15.62%	Not reported	(143)
Bevacizumab	Avastin	VEGF	Humanized IgG1	Colorectal cancer	2005/2004	0%	0%	(144)
Bezlotoxumab	Zinplava	*Clostridium difficile enterotoxin B*	Human IgG1	Prevention of Clostridium difficile infection recurrence	2017/2016	0%	0%	https://www.accessdata.fda.gov/drugsatfda_docs/nda/2016/761046Orig1s000ClinPharmR.pdf
Brodalumab	Siliq, LUMICEF	IL-17R	Human IgG2	Plaque psoriasis	2017/2017	2.70%	0%	(145)
Burosumab	Crysvita	FGF23	Human IgG1	X-linked hypophosphatemia	2018/2018	0%	0%	https://www.ultragenyx.com/file.cfm/29/docs/Crysvita_Full_Prescribing_Information.pdf
Canakinumab	Ilaris	IL-1β	Human IgG1	Muckle-Wells syndrome	2009/2009	<1%	0%	https://www.accessdata.fda.gov/drugsatfda_docs/nda/2016/125319Orig1s085,086,087MedR.pdf
Cemiplimab	Libtayo	PD-1	Human mAb	Cutaneous squamous cell carcinoma	2019/2018	1.30%	Not reported	https://www.accessdata.fda.gov/drugsatfda_docs/label/2018/761097s000lbl.pdf
Cetuximab	Erbitux	EGFR	Chimeric IgG1	Colorectal cancer	2004/2004	22.36%	Not reported	(90)
Crizanlizumab	Adakveo	CD62 (aka P-selectin)	Humanized IgG2	Sickle cell disease	In review/2019	0–1.6%	0%	https://www.accessdata.fda.gov/drugsatfda_docs/label/2019/761128s000lbl.pdf
Daratumumab	Darzalex	CD38	Human IgG1	Multiple myeloma	2016/2015	0.70%	Not reported	https://www.ema.europa.eu/en/documents/variation-report/darzalex-h-c-4077-ii-0002-epar-assessment-report-variation_en.pdf
Denosumab	Prolia	RANK-L	Human IgG2	Bone Loss	2010/2010	0%	0%	(146)
Dinutuximab	Unituxin	GD2	Chimeric IgG1	Neuroblastoma	2015/2015	28%	Not reported	(147)
Durvalumab	IMFINZI	PD-L1	Human IgG1	Bladder cancer	2018/2017	2.90%	Not reported	https://www.accessdata.fda.gov/drugsatfda_docs/label/2018/761069s002lbl.pdf
Eculizumab	Soliris	C5	Humanized IgG2/4	Paroxysmal nocturnal hemoglobinuria	2007/2007	0%	0%	(148)
Elotuzumab	Empliciti	SLAMF7	Humanized IgG1	Multiple myeloma	2016/2015	33.30%	Not reported	(149)
Emapalumab, emapalumab-lzsg	Gamifant	IFNg	Human IgG1	Primary hemophagocytic lymphohistiocytosis	In review/2018	5%	1.60%	https://www.accessdata.fda.gov/drugsatfda_docs/nda/2018/761107Orig1s000MultidisciplineR.pdf
Erenumab	Aimovig	CGRP receptor	Human IgG2	Migraine prevention	2018/2018	8.90%	0%	https://www.accessdata.fda.gov/drugsatfda_docs/nda/2018/761077Orig1s000SumR.pdf
Evolocumab	Repatha	PCSK9	Human IgG2	High cholesterol	2015/2015	0.16%	0%	(150)
Evolocumab	Dupixent	IL-4R α	Human IgG4	Atopic dermatitis	2017/2017	2–6%	4–9%	https://www.accessdata.fda.gov/drugsatfda_docs/label/2018/761055s007lbl.pdf
Fremanezumab	Ajovy	CGRP	Humanized IgG2	Migraine prevention	2019/2018	0.4–1.6%	0.06–0.9%	https://www.accessdata.fda.gov/drugsatfda_docs/label/2018/761089s000lbl.pdf
Galcanezumab	Emgality	CGRP	Humanized IgG4	Migraine prevention	2018/2018	12.50%	Most ADA were ntADA	https://www.accessdata.fda.gov/drugsatfda_docs/nda/2018/761063Orig1s000ClinPharmR.pdf
Golimumab	Simponi	TNFa	Human IgG1	Rheumatoid and psoriatic arthritis, ankylosing spondylitis	2009/2009	31.70%	Not reported	(151)
Guselkumab	TREMFYA	IL-23 p19	Human IgG1	Plaque psoriasis	2017/2017	5.50%	0.40%	https://www.accessdata.fda.gov/drugsatfda_docs/nda/2017/761061Orig1s000MultidisciplineR.pdf
Ibalizumab, ibalizumab-uiyk	Trogarzo	CD4	Humanized IgG4	HIV infection	2019/2018	0.83%	0.83%	https://www.accessdata.fda.gov/drugsatfda_docs/nda/2018/761065Orig1s000ClinPharmR.pdf
Infliximab	Remicade	TNF	Chimeric IgG1	Crohn’s disease	1999/1998	66.70%	Not reported	(139; 152)
Ipilimumab	Yervoy	CTLA-4	Human IgG1	Metastatic melanoma	2011/2011	26%, 1.1–5.4%	Not reported, 0%	(153), United States Product Information 2018
Ixekizumab	Taltz	IL-17a	Humanized IgG4	Psoriasis	2016/2016	9%	Not reported	(154)
Lanadelumab	Takhzyro	Plasma kallikrein	Human IgG1	Hereditary angioedema attacks	2018/2018	12%	Not reported	https://www.accessdata.fda.gov/drugsatfda_docs/label/2018/761090s000lbl.pdf
Mepolizumab	Nucala	IL-5	Humanized IgG1	Severe eosinophilic asthma	2015/2015	3%	<1%	(155)
Mogamulizumab	Poteligeo	CCR4	Humanized IgG1	Mycosis fungoides or Sézary syndrome	2018/2018	3.90%	0%	https://www.accessdata.fda.gov/drugsatfda_docs/nda/2018/761051Orig1s000MultidisciplineR.pdf
Natalizumab	Tysabri	a4 integrin	Humanized IgG4	Multiple sclerosis	2006/2004	8–9%	Not reported	(156)
Necitumumab	Portrazza	EGFR	Human IgG1	Non-small cell lung cancer	2015/2015	4.10%	1.40%	https://www.accessdata.fda.gov/drugsatfda_docs/label/2015/125547s000lbl.pdf
Nivolumab	Opdivo	PD1	Human IgG4	Melanoma, non-small cell lung cancer	2015/2014	12.7%, 4.1–37.8%	0.8%, 0–4.6%	(157) https://www.accessdata.fda.gov/drugsatfda_docs/label/2019/125554s070lbl.pdf
Obiltoxaximab	Anthim	*B. anthracis* PA	Chimeric IgG1	Prevention of inhalational anthrax	In review/2016	0%	0%	(158)
Obinutuzumab	Gazyva, Gazyvaro	CD20	Humanized IgG1	Chronic lymphocytic leukemia	2014/2013	7%	Not reported	https://www.accessdata.fda.gov/drugsatfda_docs/label/2017/125486s017s018lbl.pdf
Ocrelizumab	OCREVUS	CD20	Humanized IgG1	Multiple sclerosis	2018/2017	0.9%, 0.2–0.5%	0.15%, 0–0.2%	https://www.accessdata.fda.gov/drugsatfda_docs/nda/2017/761053Orig1s000ClinPharmR.pdf, (159)
Ofatumumab	Arzerra	CD20	Human IgG1	Chronic lymphocytic leukemia	2010/2009	<1%	Not reported	https://www.accessdata.fda.gov/drugsatfda_docs/label/2016/125326s062lbl.pdf
Olaratumab	Lartruvo	PDGFRα	Human IgG1	Soft tissue sarcoma	2016/2016	3.50%	3.50%	https://www.accessdata.fda.gov/drugsatfda_docs/nda/2016/761038Orig1s000MultiDisciplineR.pdf
Omalizumab	Xolair	IgE	Humanized IgG1	Asthma	2005/2003	0%	0%	(160)
Palivizumab	Synagis	RSV	Humanized IgG1	Prevention of respiratory syncytial virus infection	1999/1998	1.80%	0%	(161)
Panitumumab	Vectibix	EGFR	Human IgG2	Colorectal cancer	2007/2006	4.60%	1.60%	https://www.accessdata.fda.gov/drugsatfda_docs/label/2009/125147s080lbl.pdf
Pembrolizumab	Keytruda	PD1	Humanized IgG4	Melanoma	2015/2014	1.80%	0.50%	(162)
Pertuzumab	Perjeta	HER2	humanized IgG1	Breast Cancer	2013/2012	0.60%	Not reported	(163)
Ramucirumab	Cyramza	VEGFR2	Human IgG1	Gastric cancer	2014/2014	3.80%	0.18%	https://www.accessdata.fda.gov/drugsatfda_docs/nda/2014/125477Orig1s000MedR.pdf
Ravulizumab (ALXN1210)	Ultomiris	C5	Humanized IgG2/4	Paroxysmal nocturnal hemoglobinuria	2019/2018	>0.5%	0%	https://www.accessdata.fda.gov/drugsatfda_docs/nda/2018/761108Orig1s000MultidisciplineR.pdf
Raxibacumab	(Pending)	*B. anthracis* PA	Human IgG1	Anthrax infection	NA/2012	0%	0%	https://www.accessdata.fda.gov/drugsatfda_docs/label/2012/125349s000lbl.pdf
Reslizumab	Cinqaero, Cinqair	IL-5	Humanized IgG4	Asthma	2016/2016	4.8–5.4%, 5%	Not reported, 0%	(164; 165)
Risankizumab	Skyrizi	IL-23 p19	Humanized IgG1	Plaque psoriasis	2019/2019	24%	14%	https://www.accessdata.fda.gov/drugsatfda_docs/nda/2019/761105Orig1s000MultidisciplineR.pdf
Rituximab	MabThera, Rituxan	CD20	Chimeric IgG1	Non-Hodgkin lymphoma	1998/1997	26–37%, 12.5%	Not reported	(73; 144)
Romosozumab	Evenity	Sclerostin	Humanized IgG2	Osteoporosis in postmenopausal women at increased risk of fracture	NA/2019	18.10%	4.60%	https://www.accessdata.fda.gov/drugsatfda_docs/nda/2019/761062Orig1s000MultidisciplineR.pdf
Sarilumab	Kevzara	IL-6R	Human IgG1	Rheumatoid arthritis	2017/2017	14–19.3%	1.8–3.3%	https://www.accessdata.fda.gov/drugsatfda_docs/nda/2017/761037Orig1s000ChemR.pdf
Secukinumab	Cosentyx	IL-17a	Human IgG1	Psoriasis	2015/2015	0.41%	0.20%	(166)
Siltuximab	Sylvant	IL-6	Chimeric IgG1	Castleman disease	2014/2014	0.20%	0%	(167)
Tildrakizumab	Ilumya	IL-23 p19	Humanized IgG1	Plaque psoriasis	2018/2018	6.8–8.8%, 4.1–8.2%	2.7–3.34%, 0.6–3.2%	https://www.accessdata.fda.gov/drugsatfda_docs/nda/2018/761067Orig1s000MultdisciplineR.pdf, (168)
Tocilizumab	RoActemra, Actemra	IL-6R	Humanized IgG1	Rheumatoid arthritis	2009/2010	5	Not reported	(169)
Trastuzumab	Herceptin	HER2	Humanized IgG1	Breast cancer	2000/1998	16.30%	Not reported	(144)
Ustekinumab	Stelara	IL-12/23	Human IgG1	Psoriasis	2009/2009	6.50%	Not reported	(170)
Vedolizumab	Entyvio	α4β7 integrin	humanized IgG1	Ulcerative colitis, Crohn’s disease	2014/2014	17%	Not reported	(171)

In the past decade, next-generation sequencing (NGS) technologies enabled a rapid increase in the capacity to sequence human and animal genomes (32). Like many other areas of modern biology, NGS is now frequently used in basic and applied immunology. NGS is often applied for sequencing the V_*H*_ and V_*L*_ antibody domains (33–36), as well as T-cell receptors (37, 38) and antibody derivative [e.g., scFv, F(ab)] libraries screened using display systems (39–41). NGS analysis of B cells can elucidate the features of antibody immune responses at a molecular level, and has been further exploited for advanced mAb discovery and engineering (42–44).

In addition to NGS of bulk populations, single-cell sequencing comprises an important group of technologies for antibody discovery, as single cell data is necessary to reveal the native V_*H*_ and V_*L*_ pairing. Previous studies were able to obtain V_*H*_ and V_*L*_ chain pairing from isolated plasmablasts (PB) in immunized mice (34, 45, 46) and antigen-specific PB from tetanus-vaccinated human patients (33).

A recently introduced technology combines proteomic analyses of antibodies in blood or secretions with NGS analysis of antibody-encoding B cells. Proteomics thus provides invaluable information about the molecular, monoclonal properties of human serum antibodies in health and disease (46–48). All of the above recently developed technologies have expedited mAb discovery and revolutionized our understanding about the nature of the immune responses, including in the formation of ADAs following immunization and administration of mAbs.

Monoclonal antibodies immunogenicity is mainly manifested in ADA generation (49). The formation of ADAs alters a drug’s bioavailability and pharmacokinetic and pharmacodynamic properties, and most often reduces drug efficacy (50, 51). ADAs have a significant impact on mAb drug safety, as they can lead to serious adverse immune reactions in the clinic (52). Patients with ADAs can be stratified by their effect on the clinical treatment course. Patients are designated as having primary loss of response (LOR) when the administrated mAb fails to show any efficacy within several weeks following treatment initiation, or secondary LOR when patients show significant side effects or the drug loses effectiveness over time despite an initial therapeutic response (53–55).

For multiple decades, many studies focused on possible mechanisms that govern ADA formation, development of improved assays for ADA detection, and advancement of tools for immunogenicity and prediction of ADA formation. This review provides an overview on these topics, underlining the challenges and potential solutions for this important research field. While this review focuses on ADA as an important outcome of mAb immunogenicity, there are other immunogenicity outcomes such as allergic reactions, cytopenia, and anaphylaxis that are widely reviewed elsewhere (56).

## The Molecular Mechanisms That Lead to ADA Formation

Anti-drug antibodies can be generated by a T-cell dependent or independent B cell activation pathway. In the T-cell dependent pathway, mAbs act as antigens and are internalized by antigen presenting cells (APCs), processed, and presented to T cells via the cognate interaction between the MHC class II molecules and T-cell receptor. Depending on the cytokine milieu during this interaction, several different immune responses can occur (57). In the T-cell dependent pathway, ADAs are generated when a T helper cell (Th) differentiates into a Th1 or Th2 phenotype and, following their cognate interactions with B cells, induces the proliferation of plasma cells (PC) that secrete ADAs. Previous studies showed that a Th2 response mostly induce ADA production of the IgG4 isotype, in comparison to the Th1 response, that in the case of anti-factor VIII elicits the generation of IgG1 and IgG2 ADA (58, 59).

For example, infliximab-specific Th2 cells can be detected in circulation after infliximab infusion, and these cells were correlated with the presence of infliximab-specific ADA (60). Interestingly, this cellular response was observed mostly in patients with hypersensitivity reactions, rather than in the LOR group. In another study, T cell epitopes of infliximab and rituximab were identified by isolating antibody-specific T cells after repeated rounds of antibody-loaded dendritic cells (DCs) in co-culture (61). These T cells were specific to peptides derived from V_*H*_ and V_*L*_ and encompassed CDRs and FRs, reflecting the immunogenicity of the chimeric part of these antibodies. Importantly, these peptides were also eluted from antibody-loaded DCs, highlighting the importance of MHC Class II antigen presentation in the ADA formation process.

In contrast, for the T cell independent pathway mAbs with multiple epitopes can crosslink B cell receptors (BCRs) and stimulate B cells to differentiate into PC to produce ADAs (62–66). It was previously demonstrated that impurities and aggregates of the mAbs may increase the number of adjacent epitopes on the mAb, potentially steering the immune response toward a T-cell independent pathway by B cell crosslinking (67–70).

## Drug and Patient Characteristics Contributing to ADA Formation

**FIGURE 1:**
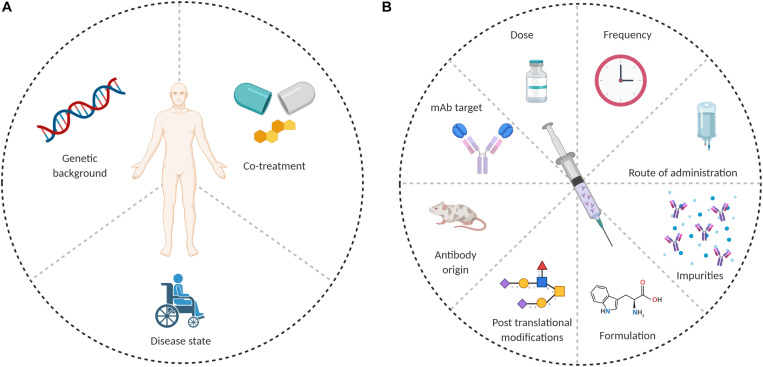
Possible causes of ADA formation. **(A)** Patient related and **(B)** drug related.

Anti-drug antibodies formation depends on the interplay between several factors, which can be patient-related or drug-related. Possible causes for ADA formation are summarized in Figure 1.

### Patient-Related Factors

The study of why and how ADAs are generated is complicated by the fact that some patients develop ADAs and some, with the same clinical indication and receiving the same therapeutic mAb, do not. The extent of immunogenicity thus differs among patients receiving the same mAb, which could be related to the immune pathways underlying the pathogenesis of the disease (71). For example, RA patients have a higher likelihood of developing ADAs toward a mAb drug than spondyloarthritis patients (57). When examining a specific disease or immune target, different mAbs may have a varying effect on the induction of ADAs. RA patients develop higher ADA levels when treated with two different mAbs (72). In multiple sclerosis (MS) patients, treatment with rituximab (chimeric anti-CD20 mAb) generated an unwanted immune response in up to 37% of patients (73). On the contrary, belimumab (a fully human anti B-cell activating factor (BAFF) mAb), which is used to treat systemic lupus erythematosus (SLE) patients, showed low rates induction ADA (74). Of note, in autoimmune diseases the hyperactivation of both the innate and adaptive immune responses may further complicate the study of mAb immunogenicity (57, 75). On the other hand, when administering mAbs to cancer patients, ADA formation often depends on the stage of the cancer. ADA levels tend to be higher in early stages of the disease than in later stages (76).

Much of the variability in the propensity of administrated mAb to induce ADA formation may result from different immune contexts; Principally, disease status and HLA alleles, which could promote or inhibit an ADA response. The idea that ADA formation is often derived from a T-dependent response has recently led to studies focusing on how ADA formation correlates with HLA polymorphism in the population. Although limited by sample size, Benucci et al. showed that patients with the HLA-DRβ-11, HLA-DQ-03, and HLA-DQ-05 alleles were at a higher risk to develop ADA responses after treatment with an anti-TNF mAb (5 different mAbs were included in this study) (77). Another report revealed that a G1m1 allotype in the IgG1 created a protease cleavage site in the CH3 domain of the antibody Fc and enabled presentation of a CH3_15–29_ peptide epitope (78). The CH3_15–29_ peptide epitope was tolerated in patients with a G1m1 allotype. However, donors homozygous for nG1m1 did not natively display the G1m1 MHC-II peptide and developed T cell CD4^+^ responses against antibody therapeutics containing the G1m1 allotype sequence; these ADA were also correlated with HLA-DRB1^∗^07 allele. Some therapeutic mAbs (including trastuzumab) do not harbor this allotype, which could partially explain differences in immunogenicity across different mAb drugs (78, 79). This allotype difference could impact future development of antibody products, since ∼40% of the Caucasian population is homozygous for nG1m1, and thus may be at a greater risk for ADA generation (80). In two recent studies, ADA formation against infliximab and adalimumab was correlated with the HLADQA1^∗^05A > G genotype in IBD patients (81, 82). One detailed recent study examined the immune response to natalizumab, a humanized monoclonal IgG4 antibody to α4 integrins that is used to treat patients with MS, and that induces ADA formation in ∼6% of the patients. The immune response was found to be polyclonal and targeted different epitopes of the natalizumab idiotype, with a single immunodominant T cell epitope spanning the FR2-CDR2 region of the V_*L*_ (83). Generation of a T cell-dependent ADA response is also a multifactorial process, depending not only on the existence of a potential MHC-II peptide epitope in the mAb, but also on the ability of that epitope to be processed, presented and recognized by T cells. The influence of HLA allotypes on the probability of ADA responses should be considered during the design of immunogenicity studies and clinical trials for mAb development. Conclusions from studies that rely on smaller cohorts might not have general applicability for ADA predictions if the study population has substantially different MHC-II gene backgrounds from a larger treatment population.

### Drug-Related Factors

The molecular mechanisms that lead to induction of ADAs were initially related to the murine origin of the first mAbs, which were recognized as “non-self” by the human immune system. Unfortunately, even the use of complete human antibody genes has not completely eliminated immunogenicity and the associated induction of ADA (84). Fully human mAbs contain new epitopes in the CDRs that can steer the immune response through an idiotype/anti-idiotype interaction (85, 86). As discussed above, mAb-derived peptides presented by MHC-II are necessary for T cell-dependent ADA formation. Efforts to remove T cell epitopes during mAb engineering are used consistently, but the high genetic variability of human populations greatly complicates efforts to remove all MHC-II-binding peptides from human mAbs (87, 88).

Changes in Fc glycosylation may also affect ADA induction. The removal of N-linked glycosylation of the Fc was shown to reduce immunogenicity (89). Fully human mAbs lacking Fc functions were also shown to be immunogenic and have direct effects on the ability to recruit macrophages and activate complement. For example, galactose-α-1,3-galactose, which is a foreign glycan not found in humans, is present on the antigen-binding (Fab) portion of the cetuximab V_*H*_ (a chimeric mAb used in cancer therapy targeting the EGF receptor). This glycan was shown to induce ADA formation of the IgE isotype, and was responsible for anaphylactic reactions in patients (90, 91). On the other hand, immunogenicity is sometimes linked to impurities in the formulation process, and not necessarily due to glycosylation differences. A review of the differences between 18 biosimilars and mAbs originators concluded that the differences between them are mainly in glycosylation patterns, and do not impact immunogenicity (92).

Other drug related factors that play a role in mAb immunogenicity are “danger signals” that are released by tissues undergoing stress, damage or abnormal death. The danger model was first suggested in 1994, were it was first postulated that the immune system responds to substances that cause damage, rather than to those that are simply foreign (93, 94). In the case of therapeutic antibodies, process related impurities (such as aggregates and residual DNA or proteins from the mAb expression system) can influence immunogenicity (95).

The mAb target may also have high importance for the MOA of ADA formation. We recently found that repeated administration of infliximab (a TNFα antagonist) results in a vaccine-like response, where ADA formation is governed by the extrafollicular T cell-independent immune response (96). The administration of infliximab blocks TNFα and shifts the immune response toward the marginal zone (MZ) instead of the germinal center (GC), as observed in TNFα knockout mice (97). Another possible explanation is that a strong T cell-independent immune response in the MZ may be induced by a drug/ADA/TNFα immunocomplex (IC). As a trimer, TNFα may form “super complexes” upon engagement with TNFα antagonistic antibodies (98–100).

Another example of mAb target importance is alemtuzumab, a mAb specific to the CD52 lymphocyte cell surface glycoprotein. Alemtuzumab is used to treat MS (101) and induces ADAs in about 85% of patients, of which around 92% develop neutralizing ADAs (102). Alemtuzumab’s high frequency of ADA induction may be related to CD52 expression patterns. Alemtuzumab targets APCs, which include DCs, monocytes, and memory B cells, based on their CD52 expression. When monocytes repopulate, they encounter the circulating mAb that rapidly presents antigen to the antigen-specific lymphocytes (103, 104). Memory B cells often exhibit homeostatic expansion following treatment with alemtuzumab (105), which could complement ADA generation.

mAb dosage and schedule are other possible factors influencing ADA formation rates. Increased numbers of injections and higher mAb doses are associated with higher ADA risk, although some cases of chronic treatment and higher doses have lower immunogenicity (92, 106). For example, rituximab, a chimeric mAb anti-CD20, targets surface antigens on pre-B cells and B cells before their differentiation into PCs. As rituximab selectively depletes CD20 positive B cells, it does not affect mature PCs and does not have a propensity to elicit ADAs (107).

## Assays for Immunogenicity Assessment and Tools for Immunogenicity Reduction

### Pre-clinical Setting

Due to the growing importance of mAb immunogenicity, there has been a growing need for tools to assess immunogenicity and reduce the propensity of mAbs to induce ADAs. Great efforts in tools such as *in silico* prediction algorithms and cell based experimental assays are facilitating immunogenicity assessment, especially during the initial development phases of the mAb (108).

*In silico* CD4^+^ T cell epitope prediction models are often used to identify potentially immunogenic MHC-II peptide epitopes. These algorithms are based on the affinity of mAb-derived peptides to MHC-II (109–111).

With recent advances in proteomics and sequencing, several MHC-II peptide epitope databases have been constructed that provide a library of MHC-II binding data to enable immunogenicity prediction (112). Most algorithms that predict the immunogenic sequences recognized by T cells are later confirmed by assessing peptide binding to MHC molecules (88, 113). For example, a strong correlation was found between *in silico* evaluation of T cell epitopes from a recombinant Fc fusion protein, and the immunogenicity rate when administered to patients in a clinical trial (114). While such predictive algorithms are common used, they capture only a fraction of the system’s complexity. Most CD4 + T cell epitope prediction algorithms are based on binding affinity and stability to MHC-II molecules (88, 110), but fail to consider other essential factors in the recognition of T cell epitopes. Among these factors are protease cleavage sites (115), T cell precursor frequency (116), and peptide and T cell competition (117).

Experimental tools are also used to make pre-clinical predictions about mAb immunogenicity risk. These include HLA binding assays, DC related assays, T cell stimulation assays, peripheral blood mononuclear cell (PBMC) stimulation assays, and various animal models (115). HLA binding and DC antigen presentation assays can evaluate potential T cell epitopes derived from the mAb, while T cell and PBMC stimulation assays examine whether a mAb can activate immune cells *in vitro* and *ex vivo* in terms of cell proliferation and cytokine release. For example, T cell epitopes in the variable regions of infliximab and rituximab were able to stimulate peripheral blood mononuclear cells (PBMCs) to secrete a variety of cytokines (61). In another study, the immunogenicity of secukinumab, an anti- interleukin-17A mAb used to treat plaque psoriasis, was assessed by examining T-cell proliferation (118).

Each of these experimental tools has limitations in assessing and predicting immunogenicity. While considered reliable and straightforward, most of the experimental assays are labor intensive and are impractical to implement with a large number of mAb candidates. These assays are often performed with cells derived from a naïve population, where the frequency of antigen-specific cells is relatively low and precludes a clear positive result due to low signal-to-noise ratios (88).

Other advancements are being made in the development of mAbs to which patients will be more tolerant. A previous study identified a set of naturally occurring human regulatory T cell epitopes (“Tregitopes”), present in the Fc and Fab domains of IgG, that induce tolerance when co-administered with other proteins (119). When incubated with PBMCs *in vitro*, Tregitopes activated CD4^+^ T cells and increased expression of regulatory cytokines, chemokines, and CD25/Foxp3. When were administered *in vivo* with protein antigens, Tregitopes inhibited T cell proliferation, reduced effector cytokine expression, and induced antigen-specific adaptive tolerance. Co-administration of Tregitopes along with mAbs may be a useful tool for tolerization of mAbs.

### Clinical Settings

**FIGURE 2:**
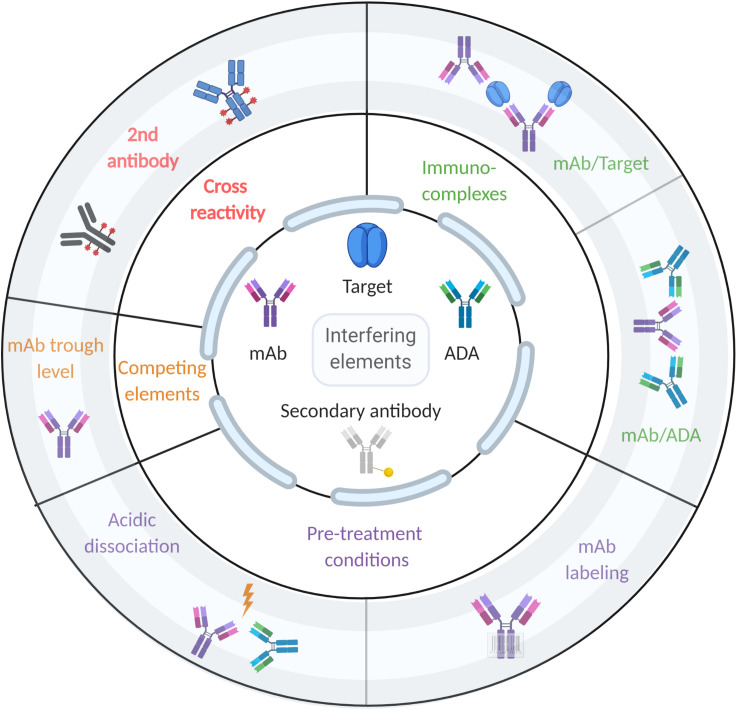
Factors that affect ADA detection in immunoassays. The center of the figure designates the components that could interfere with ADA detection (i.e., mAb, target, ADA, and secondary antibody). The middle circle designates the type of interference, while the outer circle provides examples of such interferences.

Early and accurate ADA detection is extremely important for patients treated with biologics, especially for mAbs (120). ADA detection is required to provide the clinicians with sufficient information to monitor treatment and determine optimal intervention strategies (121). Detection of ADA against therapeutic mAbs is highly challenging since both the drug and the analyte are antibodies. Moreover, immunoassays are prone to biases due to the presence of the drug and immune-complexes in patients’ serum. Historically, studies of the response following mAb administration and ADA prevalence have been inconsistent, partly due to the various assay formats used to monitor immunogenicity in clinical trials (122). Each available format has its limitations that can reduce the assay’s utility in clinical and research settings, and also complicate interpretation of the data. Some assays have poor dynamic range and may generate false-negative results because of interfering interactions with the active drug, or false-positive results due to other antibodies like rheumatoid factor (123). Figure 2 shows the competing factors which affect accurate measurement of ADAs.

An ELISA-based bridging assay is one of the most commonly used assays for ADA screening, where the mAb drug is used to first capture ADA present in the patient sera, and the latter are detected by adding additional labeled mAb as a secondary probe. Bridging ELISA assays are used for ADA detection of a large variety of mAbs, and some include an acidic step to dissociate ADA from the mAb. The excess mAb is then captured or removed, and free ADA can be detected. These assays often have significantly higher background and suffer from low sensitivity due to the disassociation of antibodies. Bridging assays can also result in false-negatives, as they are more likely to “miss” low affinity IgM ADAs present in early stages of the immune response (124). Most ELISA-based bridging assays are also sensitive to the mAbs’ trough levels (levels of circulating mAb at sampling time). ADA and mAbs tend to form high molecular weight immune-complexes, making ADA detection more challenging (125). To overcome this challenge, several drug-tolerant assays have been developed to measure ADA levels in the presence of high mAb concentrations (126). Most of these assays also use an acidic treatment step. Several other techniques have been reported to evaluate serum ADA levels. These assays include radio-immunoassays (127), Biotin-drug Extraction with Acid Dissociation (BEAD) (128), Precipitation and Acid dissociation (PANDA) (129), Affinity Capture Elution ELISA (ACE) (130), and Homogenous Mobility Shift Assay (HMSA) (131); these assays have been reviewed in detail elsewhere (126). While these assays presumably detect all serum ADA, they primarily provide qualitative measures to assist healthcare providers deciding on appropriate patient interventions, and many (if not all) studies underestimate actual ADA levels. These assays also lack standardization that could enable comparisons of ADA levels across health centers. The great diversity in these assays poses tremendous difficulty in studying ADA levels between different mAbs, across studies of the same mAb, and across different assays.

In a clinical context, it important both to assess ADA levels in patient serum, and also to assess the presence of neutralizing antibodies that interfere with biological and clinical activity of the mAb. The neutralizing effect of ADAs can be assayed by testing whether ADAs in serum inhibit binding of the mAb to its target (132). Several cell-based assays were developed to detect *nt*ADA in patients’ serum. One of these assays is a functional ADA cell-based assay that was developed to quantify the activity of TNFα antagonists. This assay assesses both drug activity and *nt*ADA levels (133), but correlations between the clinical outcome and assay results were not thoroughly tested. Another assay developed for *nt*ADA detection is the reporter gene assay, which is based on excretion of IL8 by HT29 cells due to TNFα stimulation (77). When the assay was applied to sera samples with low-level ADA, it detected *nt*ADA even prior to clinical LOR to the mAb, which allows the prediction of clinical LOR with high probability.

While these assays are accurate and sensitive, they require an active cell line, which complicates assay implementation. We recently reported on a newly developed quantitative bio-immunoassay for quantifying ADA specific to TNFα antagonists. The bio-immunoassay was further modified to easily assess the neutralization capacity of ADA using an *in vitro* assay (96). This assay can be readily used in a clinical setting that performs routine ADA measurements.

Other clinical approaches to reduce immunogenicity include active interference of the T cell responses to mAbs, thereby inducing individual tolerance of the immune system (“tolerization”).

For example, administration of methotrexate (MTX) with infliximab reduced ADA formation in RA patients (134). MTX also reversed high ADA levels in infantile Pompe disease patients treated with rituximab, when administered alongside bortezomib, a proteasome activity inhibitor that leads to cell death (135). Azathioprine is also an immunosuppressive drug that can be given in combination with infliximab or adalimumab to improve treatment and reduce immunogenicity and ADA formation (136–138). However, such non-specific immunosuppressive approaches have potentially harmful side effects that must be balanced with the patient’s overall treatment plan.

## Concluding Remarks

Monoclonal antibodies have the potential to treat a wide range of diseases and disorders, but they can be highly immunogenic and induce undesirable ADA responses. ADAs can reduce mAb drug efficacy by altering its bioavailability and/or accelerating clearance from circulation. While the molecular mechanisms of ADA generation are not fully understood, it is dependent on both patient and drug characteristics. While early ADAs were related to the murine origin of the first mAb therapeutics, ADAs also occur against fully human mAbs. Indeed, complete humanization cannot completely abrogate mAb immunogenicity and ADA formation. The questions of why and how ADA are generated also depend on variability of the reported immunogenicity rates, which emphasizes the need for standardized clinical assays for ADA detection. Understanding the mechanisms of ADA generation and the major factors that influence immunogenicity of mAbs will help us design safer mAbs with lower drug rejection rates. Recent and ongoing efforts to study mAb immunogenicity at the molecular level is augmenting our understanding of these mechanisms that lead to ADA formation, which may help provide new guidelines to improve the safety and efficacy of mAb therapeutics.

## Author Contributions

AV-M, MG-G, BD, and YW wrote the sections of the manuscript. All authors contributed to manuscript revision, read, and approved the submitted version.

## Conflict of Interest

The authors declare that the research was conducted in the absence of any commercial or financial relationships that could be construed as a potential conflict of interest.
